# In-Silico Analysis of Phytocompounds of *Olea europaea* as Potential Anti-Cancer Agents to Target PKM2 Protein

**DOI:** 10.3390/molecules27185793

**Published:** 2022-09-07

**Authors:** Faizan Abul Qais, Suliman Yousef Alomar, Mohammad Azhar Imran, Md Amiruddin Hashmi

**Affiliations:** 1 Department of Agricultural Microbiology, Faculty of Agricultural Sciences, Aligarh Muslim University, Aligarh UP-202002, India; 2 Department of Zoology, College of Science, King Saud University, Riyadh 11451, Saudi Arabia; 3 Department of Internal Medicine, Gangnam Severance Hospital, Yonsei University College of Medicine, Seoul 06273, Korea; 4 Interdisciplinary Biotechnology Unit, Aligarh Muslim University, Aligarh UP-202002, India

**Keywords:** pyruvate kinase M2 (PKM2), virtual screening, ADMET analysis, molecular docking, molecular dynamic simulations

## Abstract

Globally, cancer is the second leading cause of mortality and morbidity. The growth and development of cancer are extremely complex. It is caused by a variety of pathways and involves various types of enzymes. Pyruvate kinase M2 (PKM2) is an isoform of pyruvate kinase, that catalyses the last steps of glycolysis to produce energy. PKM2 is relatively more expressed in tumour cells where it tends to exist in a dimer form. Various medicinal plants are available that contain a variety of micronutrients to combat against different cancers. The phytocompounds of the olive tree (*Olea europaea)* leaves play an important role in inhibiting the proliferation of several cancers. In this study, the phytocompounds of olive leaf extract (OLE) were studied using various in silico tools, such as pkCSM software to predict ADMET properties and PASS Online software to predict anticancer activity. However, the molecular docking study provided the binding energies and inhibition constant and confirmed the interaction between PKM2 and the ligands. The dynamic behaviour, conformational changes, and stability between PKM2 and the top three hit compounds (Verbascoside (Ver), Rutin (Rut), and Luteolin_7_*O*_glucoside (Lut)) are studied by MD simulations.

## 1. Introduction

Cancer is initiated by alterations in the human genome that enhance cellular proliferation through variation in normal pathways and mechanisms. However, cancer is the second prominent cause of death worldwide [1]. The development of cancer is an extremely complex process, therefore, it is recognized as a multifactorial disease [2]. The main difference between cancer and normal cells is that, even when there is adequate oxygen available in the surroundings, cancer cells are completely dependent on aerobic glycolysis to generate energy. During glucose metabolism, pyruvate kinase catalyses the last step of the conversion of phosphate unit from phosphoenolpyruvate to ADP to form the end-products as pyruvate and ATP. Under aerobic conditions, tumour cells continue to convert glucose to lactate, and this phenomenon is termed the Warburg effect [3]. In mammals, there are four main types of pyruvate kinase (PK) isomers, which include PKL, PKR, PKM1, and PKM2. PKL is mainly expressed in the liver, intestine, and kidney, whereas PKR is primarily up-regulated in RBC [4]. PKM1 is expressed in the muscles, heart, and brain. However, PKM2 is up-regulated in tumours and embryonic tissues of proliferating cells [5]. In healthy tissues, PKM2 proteins transit between the tetrameric form to dimeric form. Moreover, in tumour cells, PKM2 exist in the dimeric form. The low catalytic activity of dimeric PKM2 increases the anabolic synthesis of protein through the pentose phosphate pathway and thus, stimulates the growth and development of cancer cells [6].

Olive tree (*Olea europaea*) leaves have been widely used in folk medicine in the European Mediterranean area, India, Arabian peninsula, and other tropical and subtropical regions for the treatment of inflammation, allergies, diarrhea, and diseases such as Alzheimer’s, chronic fatigue syndrome, and osteoarthritis [7]. The bioactive composites of olive leaf extract (OLE) contain a higher quantity and variety of microconstituents than extra virgin olive oil (Table 1). There is also a structural difference between the phytocompounds of OLE that shows a great enhancement to health outcomes compared with olive fruit [8]. It has been proposed that the OLE plays an important protective role in inhibiting the proliferation of several cancer cell lines including pancreatic, leukaemia, breast, prostate, and colorectal [9]. There are numerous bioactive compounds of OLE that are studied for the inhibition of progression and development of different types of cancers. A study conducted on flavonoids of OLE against HeLa cervical cancer cells reported that its increasing concentrations resulted in decreased cancer cell viability. The major compounds detected in the extract were luteolin-4′-*O*-glucoside, apigenin-7-*O*-glucoside, and rutin [10]. Similarly, another study found a cytotoxic effect of OLE extract on MCF-7 breast and HepG2 hepatocellular carcinoma cells [11]. Some of the compounds of OLE have been reported to induce apoptosis and inhibit proliferation in the cancer cells due to its potential anticancer activity [12]. In this study, we utilized in-silico approaches such as (i) pharmacoinformatics to predict the toxicity, anticancer, and pharmacokinetics properties of the selected phytocompounds against PKM2, (ii) molecular docking, and (iii) molecular dynamic simulations for evaluation of the conformational changes, stability, and intermolecular interactions between PKM2 and the phytocompounds. This study aims to identify the potential lead phytocompounds from OLE to combat PKM2-related cancer in the coming future.

## 2. Materials and Methods

### 2.1. Preparation of the Phytocompounds Database

The three-dimensional structure of the phytocompounds of OLE was retrieved from PubChem (www.pubchem.ncbi.nlm.nih.gov) in the format of SDF. The molecules were then converted to PDB format using Chimera 1.14 software (California, SF, USA) [23]. The PDB file was further used for molecular docking.

### 2.2. Prediction of ADMET Properties

Computational pharmacokinetics in an in-silico approach was used to determine the time course of drug absorption, distribution, metabolism, excretion, and toxicity (ADMET). In the early stages of the selection of phytocompounds, ADMET properties support and define the efficiency and integrity of the active compounds. To estimate the ADMET properties of phytocompounds, pkCSM server was used [24]. Toxicity can be evaluated by measuring the degree of the quality of a chemical substance being poisonous to humans or animals and that can damage an organ. It is very important to evaluate the harmful effect of chemical compounds before undergoing a drug trial. Toxicity evaluation is one of the main and vital steps in the drug design process [25].

### 2.3. Prediction of Anticancer Activity

The in-silico prediction of anticancer properties of the selected phytocompounds were further studied by using a web server named PASS-Way2Drug server. The PASS (Prediction of Activity Spectra for Substances) gives p values for the similarity measures based on which anticancer activity is predicted as highly possible or less possible [26]. It can hit 65% positive results at a rate of 0.05 false-positive outcomes. Since it requires the canonical Simplified Molecular Input Line Entry String (SMILES) of compounds to be investigated, we used the PubChem server as sources of required entries.

### 2.4. Retrieval and Preparation of Protein

In this study, the 3D structure of the PKM2 (PDB ID: 3BJF) obtained by X-ray diffraction with a resolution of 2.03 Å was retrieved from RCBS, Protein Data Dank (https://www.rcsb.org/, accessed on 8 June 2022). The human PKM2 protein is a homo-tetramer with a molecular weight of 228.50 kDa. Each chain contains 518 residues. The ions, water molecules, and other ligands were removed to make a clean PDB structure. The only monomeric structure of PKM2 was taken for molecular docking and simulation studies. A detailed procedure is given in the molecular docking section.

### 2.5. Screening of Potential Lead Compounds

The active site of enzymatic proteins contains a catalytic site, where the substrate binds, and the reaction is catalysed forming the product. Mammalian PKM2 is a tetrameric protein with identical subunits. Each monomer contains one active site and is composed of three main domains—designated A, B, and C—plus a small N-terminal domain. Molecular docking is a well-known screening tool that can analyse the best binding mode of a ligand to a targeted macromolecule. The molecular docking was performed using AutoDock-Vina software (La Jolla, CA, USA) [27]. The receptor molecule (PKM2) was cleaned before preparation. The non-polar hydrogen was added and then Kollman charges were added. The spacing of the grid was set to 1 Å. The size of the grid was 60 × 72 × 92 with centre as x = 3.580, y = −18.461, and z = 27.361. The receptor was finally saved into PDBQT format using MGLTools 1.5.6. The ligand molecules were made flexible by detecting the roots to obtain the best possible conformation. The ligands were saved in PDBQT format. The analysis of molecular docking was performed using LigPlot^+^ (Cambridge, UK), Discovery Studio 2021, AutoDock Tools (La Jolla, CA, USA), and PyMOL [28,29,30].

### 2.6. Molecular Dynamics Simulations

The top three compounds, Rut, Lut, and Ver, exhibiting the highest binding affinity towards PKM2 were used for further studies using molecular dynamics (MD) simulations. For these selected compounds, the docked conformations with the lowest binding energy were taken as the initial structure of MD simulation. The MD simulations were performed on Gromacs 2018.1 package using amber99sb-ILDN force field [31,32]. The topology of the ligands was generated with Antechamber packages in AmberTools21 [33]. PKM2 alone was also simulated as a control. All systems were solvated in a triclinic box using the TIP3P water model followed by their neutralization by adding 3 chlorine ions. The energy of the system was minimized using a maximum of 50,000 steps to remove the weak van der Waals contacts. The systems were equilibrated for NVT and NPT for 1 ns, each using a V-rescale thermostat at 310 K and using Parrinello-Rahman barostat at 1.0 bar, respectively. The equilibrated structure was finally used to perform 100 ns MD simulations. Three replicates of each system were simulated, and the data are presented as averages. Each trajectory and all trajectories were subjected to periodic boundary conditions (PBC) corrections before analysis. Using Gromacs utilities, root mean square deviation (RMSD) of the backbone was calculated for initial conformation. Furthermore, root mean square fluctuations (RMSF), hydrogen bond analysis, solvent accessible surface area analysis (SASA), radius of gyration (Rg), secondary structure of protein, etc., were calculated. The principal complement analysis (PCA) was calculated using gmx covar utility of gromacs that calculates and diagonalizes the covariance matrix and a new trajectory file. The new trajectory written was then analysed by gmx anaeig in which all structures are fitted to the structure in the eigenvector file. The free energy landscape was made using gmx sham utility. The quantitative evaluation of the interaction of ligands with PKM2 was done using MM-PBSA calculation [34].

## 3. Results and Discussion

### 3.1. Prediction of ADMET Properties

The preliminary analysis of the phytocompounds of OLE was performed for its ability to cause toxicity in different models using in-silico tools. Compared with the conventional methods, computer-aided toxicity tests are fast and inexpensive methods that eliminate potentially toxic chemical compounds at an initial stage and reduce many biological experimental tests. Using the pkCSM web server, the toxicity of all compounds was predicted. The drug-induced AMES toxicity, hepatotoxicity, skin sensitization, and *Tetrahymena pyriformis* (*T. pyriformis*) toxicity, and oral rat acute toxicity of selected compounds are listed in Table 2. AMES toxicity measures the mutagenic potential of compounds [35]. Out of all tested compounds, only the hydroxytyrosol showed AMES toxicity. In hepatotoxicological assessment, five compounds namely ursolic acid, oleanolic acid, uvaol, betulinic acid, and maslinic acid, were predicted positive. Similarly, only tyrosol was found to exhibit skin sensitization properties. *T. pyriformis* is one of the most routinely used model organisms to test the toxicity of compounds [36]. All compounds showed very low or negligible toxicity in the *T. pyriformis* model of toxicity. This indicates that most of the tested compounds were not toxic to *T. pyriformis*-based toxicity. For instance, there is no inhibition of human ether-a-go-go-related gene 1 (hERG-1) K^+^ channels in this study. The hERG-1 is toxic for the heart and may produce lethal cardiac arrhythmia. Therefore, early-stage identification of putative hERG inhibitors or non-inhibitors may play an important role in reducing cardiotoxicity. Moreover, the oral rat acute toxicity of all compounds was also evaluated. In all compounds, except lucidumoside C, the oral rat acute toxicity was found to be below 3 log µg/l. Based on the toxicological assessment of selected compounds, it may be inferred that most of the phytocompounds of OLE were not found to exhibit toxicity. The ADME properties of the phytocompounds predicted by the pkCSM web server indicate relevant pharmaceutical properties for most of the compounds as shown in Table 3.

### 3.2. Prediction of Anticancer Activity

Prior to docking, the potential cellular targets of phytocompounds of OLE were also identified through the PASS Online software (http://www.way2drug.com/passonline). Using PASS Online, we only predicted the anticancer activity in which two main parameters, pyruvate kinase inhibition and antineoplastic properties, were considered. The reason for selection of these two parameters is that this study focuses on the screening of compounds against pyruvate kinase. The PASS Online sever predicts the biological activities of compounds and presents the results in the form of indices of the biological activity or inactivity [26]. There are probabilities of either presence of biological activity (Pa) or absence of biological inactivity (Pi). The predicted pyruvate kinase inhibition and antineoplastic properties of compounds of OLE are listed in Table 4. For pyruvate kinase inhibition, the Pa values of all compounds were more than Pi, showing the probability of biological activity compared with inactivity. It is interesting to note that Pa values of the top three compounds were far more than the Pi values. This shows the greater probability of these hit compounds to inhibit the pyruvate kinase. Similarly, Pa values for antineoplastic properties of selected compounds were also far more than Pi values. These two parameters indicate higher chances of anticancer activity of these compounds.

It is documented that both the extract and phytocompounds of OLE plays a protective role in the inhibition and proliferation of several cancer cell lines such as pancreatic cancer, leukaemia, breast, prostate, colorectal etc. [9]. Moreover, certain bioactive compounds of OLE have also been found to reduce the progression of some cancer types. For insurance, the flavonoids of OLE were found to be effective against HeLa cervical cancer cells and also decreased cancer cell viability. The major compounds detected in the extract were luteolin-4′-*O*-glucoside, apigenin-7-*O*-glucoside, and rutin [10]. Similarly, another study found the cytotoxic effect of OLE extract on the MCF-7 breast, HepG2 hepatocellular carcinoma cells [11].

### 3.3. Screening of Potential Phytocompounds

**Figure 1:**
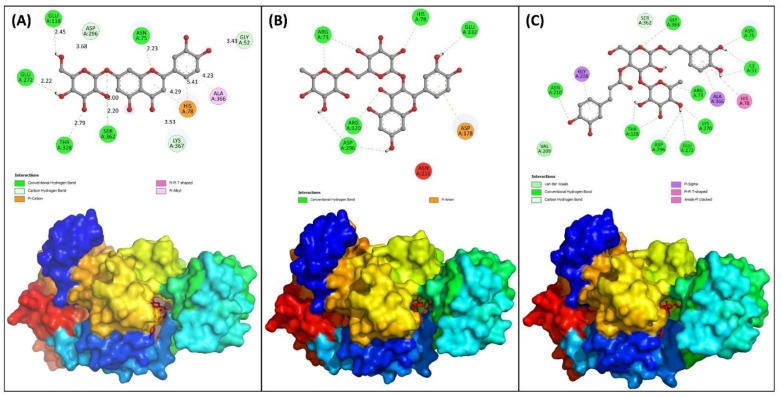
2D and 3D representation of the docked structures of the top three lead molecules showing the residues involved in the interaction of the protein-ligand complexes. (**A**) PKM2-Lut complex; (**B**) PKM2-Rut complex; (**C**) PKM2-Ver complex.

The molecular docking study was performed for virtual screening and to determine the best intermolecular interaction between the PKM2 protein and the phytocompounds. In this study, the selected phytocompounds of OLE were docked using Autodock-Vina software. The docking with Autodock-vina gave nine best conformations in which the conformation with lowest binding energy was considered. The binding energies and inhibition constant of compounds against PKM2 are listed in Table 5. Most of the compounds exhibited good binding affinity towards PKM2 and binding energies ranged from −5.7 to −10.0 kcal/mol. A total of 13 compounds were found to be docked at the catalytic site of PKM2. Only three compounds were found to bind at the allosteric site and the rest of the phytocompounds were found to interact at the other sites, i.e., sites other than catalytic and allosteric site. The top three compounds that were identified on the overall basis of binding energy, binding site, pharmacokinetics data, ADMET properties, predicted anticancer activities were Rut, Ver, and Lut. It is interesting to note that these three compounds were docked at the catalytic site of PKM2. The inhibition constant of Rut, Ver, and Lut were found to be 0.046, 0.064, and 0.296 µmol/l, respectively. The data clearly shows the potency of these compounds to inhibit PKM2 at very low concentrations. Rut interacted with Asp296, Glu332, His78, Arg120, and Arg73 of PKM2 via hydrogen bonds (Figure 1B). Asp178 formed hydrophobic interacts with Rut. Similarly, Asn210, Gly363, Asn75, Ile51, Arg73, Glu272, Lys270, Asp296, Thr328, and Ser362 formed hydrogen binds with Lut (Figure 1A). Other amino acids were involved in hydrophobic interactions. Ver was found to make hydrogen bonds with Glu118, Glu272, Thr328, Ser362, Asn75, Gly52, and Asp296 (Figure 1C). The 3D docked structure of the top three complexes are shown in Figure 1. The data clearly show the strong affinity of these compounds with PKM2 which paves the way for exploration of their therapeutic effects under in vitro and in vivo models.

### 3.4. Molecular Dynamics Simulation

#### 3.4.1. Analysis of RMSD and RMSF

**Figure 2:**
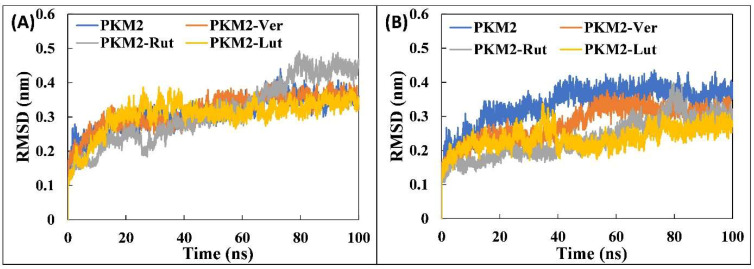
(**A**) Root mean square deviation (RMSD) of the backbone atoms of PKM2 and its complexes with Ver, Rut, and Lut over 100 ns of simulation. (**B**) RMSD of the binding pocket residues of PKM2 in the absence and presence of ligands (Ver, Rut, and Lut). The data presented are averages of three independent simulations.

**Figure 3:**
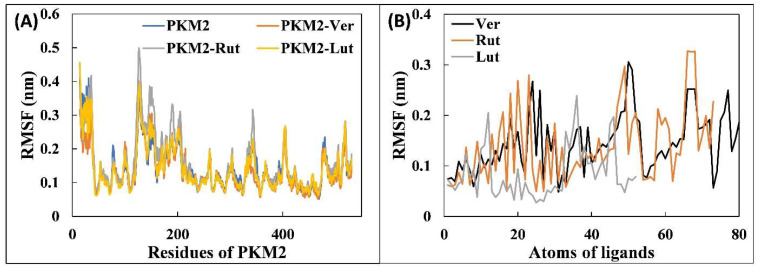
(**A**) Root mean square fluctuation (RMSF) of Cα of each residue of PKM2 and its complexes with the Ver, Rut, and Lut. (**B**) The average RMSF of each atom of ligands (Ver, Rut, and Lut). The data presented are averages of three independent simulations.

The preliminary analysis of the molecular dynamics simulation data was performed by calculating the RMSD of all systems with respect to their respective initial conformations. The RMSD values provide an idea regarding the stability of proteins/complexes during MD simulation studies. The RMSD of all systems are shown in Figure 2A. The RMSD of all systems exhibited some variations until 40 ns and then the systems became stable. This shows that all systems were well equilibrated after 40 ns. The average RMSD of PKM2 alone was found to be 0.311 nm. Similarly, the RMSD of the PKM2-Ver complex, PKM2-Rut complex, and PKM2-Lut complex was found to be 0.317, 0.313, and 0.305 nm, respectively. A slight increase in RMSD values compared with PKM2 alone is due to the dynamic behavior of ligands in the binding pocket. The RMSDs of binding pocket residues were also calculated as shown in Figure 2B. The average RMSD of the binding pocket of PKM2 alone was obtained as 0.336 nm, whereas the RMSD of the binding pocket of PKM2-Ver complex, PKM2-Rut complex, and PKM2-Lut complex were 0.279, 0.234, and 0.236 nm, respectively. It is interesting to note that the RMSD of binding pocket of all complexes decreased compared with that of PKM2 alone which is due the stabilization of the binding region after interaction of the ligands. The fluctuation in all amino acids of PKM2 alone and the complexes were studied by calculating the RMSF of C_α_ of the residues, as presented in Figure 3A. The RMSF of most of the amino acids of were found to be less than 0.25 nm, further indicating the stable nature of the systems. There were certain spikes in RMSF which are attributed to the loops and coils of PKM2 that tend to fluctuate in aqueous environments. The RMSF of each atom of all ligands were also calculated to obtain their dynamic behaviour (Figure 3B). Out of all ligands, Lut was found to exhibit the least dynamic behaviour. Other ligands, Rut and Ver, showed relatively more fluctuation indicating their dynamic behaviour in the binding pocket. This dynamic behaviour may be attributed to the rotatable bonds and the changes in interaction at binding site. As evident from the docking studies, the binding site of these three ligands were at the catalytic site of PKM2. This shows that ligands exhibited dynamical shift for their respective initial positions at the catalytic site of the protein.

#### 3.4.2. Analysis of Physicochemical Parameters, Structure Compactness and Secondary Structure

**Figure 4:**
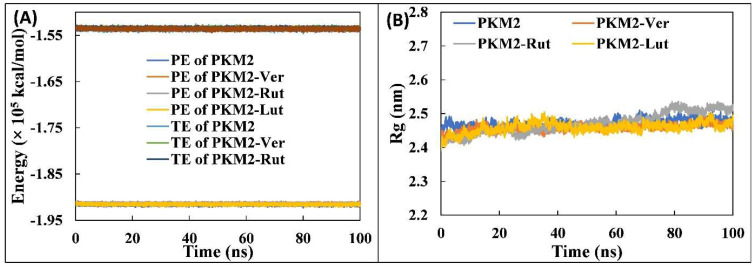
(**A**) Potential and total energy of PKM2 and its complexes with Ver, Rut, and Lut. (**B**) Radius of gyration (Rg) of PKM2 in the absence and presence of Ver, Rut, and Lut as a function of simulation time. The data presented are averages of three independent simulations.

**Figure 5:**
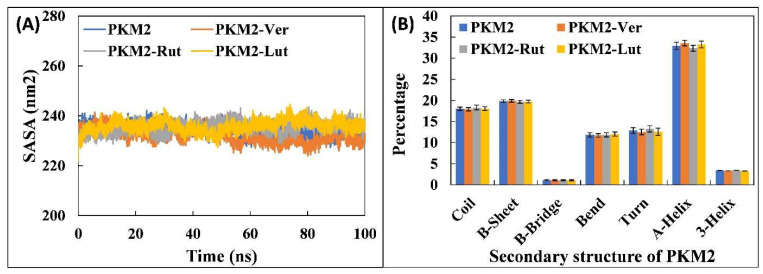
Number of hydrogen bonds formed between PKM2-Ver, PKM2-Rut, and PKM2-Lut complexes over the entire course of simulation.

Further detailed analysis of PKM2 and its complexes in aqueous environment was performed in order to validate the stability of each system and their physicochemical parameters, such as total and potential energies (Figure 4A). The potential energy of all complexes remained constant and equal to the PKM2 alone. Similarly, the total energy of all systems was found to be uniform throughout the simulation. These parameters show the stability of systems in physiological conditions [37]. The mass-weighted RMS distance of the collection of atoms from their common centre of mass is termed as radius of gyration (R_g_). Typically, compact proteins such as globular proteins have relatively lower radius variations in R_g_ compared with the expanded form of proteins [38]. The R_g_ values are taken as an important parameter to study the structural compactness and stability of proteins or complexes during MD simulation [39]. The R_g_ of PKM2 alone and its complexes are presented in Figure 4B. The average R_g_ of PKM2 alone was found to be 2.471 nm. Similarly, the R_g_ of PKM2-Ver, PKM2-Rut, and PKM2-Lut complexes were obtained as 2.458, 2.470, and 2.459 nm, respectively. The data shows the stable nature of complexes. Moreover, solvent accessible surface area (SASA) of all systems were also calculated as shown in Figure 5A. The average SASA of PKM2 alone was recorded as 234.430 nm^2^. The SASA of PKM2-Ver, PKM2-Rut, and PKM2-Lut complexes were found to be 232.062, 235.464, and 235.980 nm^2^, respectively. As evident from the data there were negligible change in the SASA of all complexes compared with PKM2 alone and the uniformity in the results further validates the stability of systems in physiological conditions.

The effect of binding of ligands on the structural stability of PKM2 was investigated by calculating secondary structure of the protein in complexed and un-complexed form. Each of the secondary structural components in PKM2 alone and complexes are shown in Figure 5B. The percentage of coils, β-Sheet, β-Bridge, bends, turns, α-helix, and 3′-helix in PKM2 alone was found to be 18.02, 19.81, 1.13, 11.81, 12.86, 32.92, and 3.42, respectively. There was no significant change in any of these motifs of PKM2 in the presence of individual ligands (Ver, Rut, and Lut). This confirms and finally validates the structural stability of all simulated complexes.

#### 3.4.3. Analysis of Hydrogen Bonds and Binding Energies

**Figure 6:**
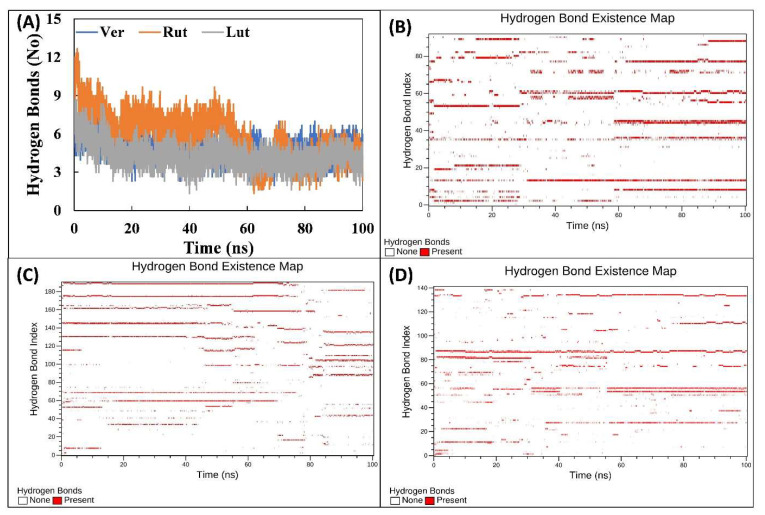
(**A**) Number of hydrogen bonds formed between PKM2-Ver, PKM2-Rut, and PKM2-Lut complexes over the entire course of simulation. The data presented are averages of three independent simulations. (**B**) The hydrogen profile for the interaction of Lut with PKM2. (**C**) The hydrogen profile for the interaction of Rut with PKM2. (**D**) The hydrogen profile for the interaction of Ver with PKM2.

To study the interaction between the ligands and PKM2, hydrogen bond profiles of all systems were calculated. First, the number of hydrogen bonds of the entire trajectories was calculated (Figure 6A). The average number of hydrogen bonds in all frames for PKM2-Ver, PKM2-Rut, and PKM2-Lut complexes were found to be 4.840, 5.801, and 4.323, respectively. This shows that Rut formed more hydrogen bonds with PKM2 than the other two ligands (Ver, Lut). The finding supports the docking studies where Rut showed the highest binding affinity towards the protein. The analysis of hydrogen bond profiles showed the existence of hydrogen bonds throughout the entire simulation period (Figure 6B–D). The shift in hydrogen bond profiles with time indicates that hydrogen bonds between the ligands and PMK2 were dynamic in nature [40]. Moreover, the fluctuation in hydrogen bonds may also be due to the interaction of water molecules at the binding site that may be resulting in fluctuation of the hydrogen bond profiles [41]. The occupancy of hydrogen bonds with more than 1.0% was also calculated. For the binding of PKM2-Ver and PKM2-Lut complexes, Asn75 showed the highest occupancy at 42.2% and 63.4%, respectively. Similarly, Glu364 of PKM2 was found to exhibit the highest hydrogen bond occupancy of 46.0% with Rut. Many other amino acids of PKM2 were also involved in the hydrogen bond formation with these ligands.

The interaction of the ligands with PKM2 was further studied using MM-PBSA calculations. A total of 100 frames from the entire trajectory of each complex at 1 ns interval was extracted for MM-PBSA analysis. In protein–ligand interactions, the non-covalent forces such as electrostatic, hydrophobic, van der Waals forces, and hydrogen bonds are predominant. These forces either contribute positively or negatively in the overall binding [42]. Various binding energies were calculated using MM-PBSA methods and the results are listed in Table 6. Electrostatic and van der Waals forces were predominant in the interaction of ligands with PKM2. There was also a small contribution of SASA energy in the total binding energy. However, polar solvation energy impaired the binding of ligands with PKM2 as evident from its positive values. The total binding energy for the interaction of Ver, Rut, and Lut with PKM2 was found to be −7.76, −3.22, and −15.00 kcal/mol, respectively.

The MM-PBSA calculations were further used to identify the major energy contributing amino acids of PKM2 (Table 7). Thr50, Pro53, Ser77, His78, Asp113, Thr114, Glu118, Val176, Asp177, Val209, Glu332, and Ala366 of PKM2 were the major energy contributors for the binding of Ver (Figure 6B–D). In the PKM2-Rut complex, Thr328, Gln329, Ile335, Gly363, and Glu364 were the major energy contributors (Figure 6C). Similarly, the binding of PKM2-Lut complex was mostly contributed by Thr50, Pro53, Leu74, His78, Gly79, Tyr83, and Ala366 residues (Figure 6D). It is interesting to note that the polar energy of some of these highest energy contributors were found to be positive, showing that the polar energies of these residues impaired the overall interaction.

### 3.5. Principal Component Analysis

**Figure 7:**
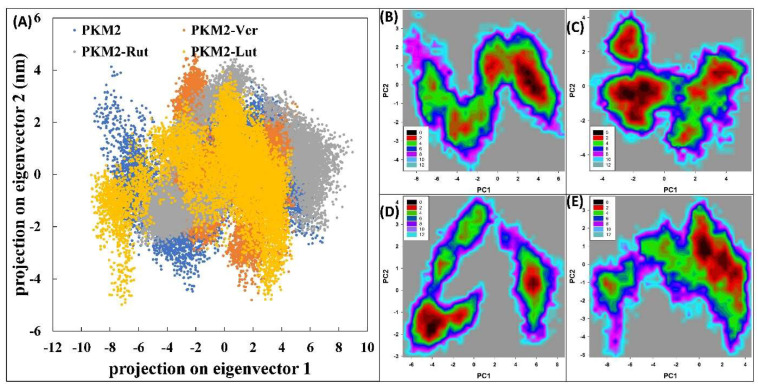
(**A**) Principal component analysis (PCA) of PKM2 in the absence and presence of Ver, Rut, and Lut. (**B**) Free energy landscape plot of PKM2 alone. (**C**) Free energy landscape plot of PKM2-Lut complex. (**D**) Free energy landscape plot of PKM2-Rut complex. (**E**) Free energy landscape plot of PKM2-Ver complex.

**Figure 8:**
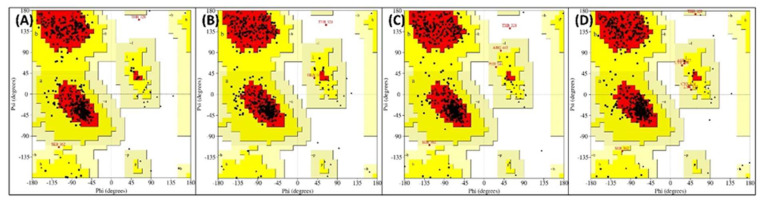
(**A**) Ramachandran plot of the energy minima structures of PKM2 alone. (**B**) Ramachandran plot of the energy minima structures of PKM2-Lut complex. (**C**) Ramachandran plot of the energy minima structures of PKM2-Rut complex. (**D**) Ramachandran plot of the energy minima structures of PKM2-Ver complex.

Principal component analysis (PCA) is one the most common standard statistical procedures to study the large-scale motion of proteins which is performed by reducing the dimensionality of data sets without losing the important information, which is characterised by eigenvectors [43]. PCA was done to analyse the differences in flexibility parameters in PKM2 and its complexes as shown in Figure 7A. All complexes and PKM alone occupied a conformational space in the projection of eigenvectors. In PCA analysis, larger conformational space denotes more structural flexibility of the proteins/complexes. This shows that the PKM2-Rut complex exhibited the highest structural flexibility compared with other complexes. The free energy landscapes were made from eigenvectors of PCA analysis to decipher the protein folding patterns. The free energy landscapes of PKM2 alone and complexes are presented in Figure 7B–E. The landscapes showed that all systems reached energy minima during MD simulation. However, there were certain differences in the position of energy minima showing the variations in their conformation. The lowest energy minima of all systems were used to extract the lowest energy structures from the respective trajectories. The Ramachandran plots for lowest energy structures are shown in Figure 8A–D. The average phi (φ) and psi (ψ) angles for PKM2 alone were found to be −77.93 and 36.58 degrees. Similarly, the average φ & ψ angles for PKM2-Ver, PKM2-Rut, and PKM2-Lut complexes were obtained as −77.16 & 35.55, −77.35 & 37.19, and −79.58 & 38.70, respectively. The negligible differences between these values show that energy minima structures of all systems were approximately equally stable, and PKM2 did not undergo any major structural transformation after the binding with the ligand molecules. A slight variation in these dihedral angles of complexes compared with PKM2 alone denotes a slight dynamical shift in the conformation of PKM2 after the interaction of ligands.

## 4. Conclusions

Medicinal plants have played an important role in the discovery of approximately 50% of anticancer drugs. The phytocompounds of OLE have a protective effect against cancer in humans. This study intends to explore the potential lead molecules from OLE as PKM2 protein inhibitors against cancers by using in-silico approaches. The top three OLE phytocompounds, Ver, Rut, and Lut, have higher binding affinities with PKM2 proteins in docking. The inhibition constant of these top three phytocompounds against PKM2 were recorded as 46, 64, and 296 nmol/L. The molecular dynamics simulations show that the ligands exhibited dynamical shift for their specific initial positions at the catalytic site of the protein. These data clearly support the strong affinity of these compounds with PKM2 that needs to be further tested under in vitro and in vivo models. The ADME properties of the top three phytocompounds, predicted using the pkCSM web server, indicated relevant pharmaceutical properties. Moreover, the PASS Online software shows the prodigious probability of hit compounds to inhibit the protein and their antineoplastic properties also indicate elevated possibilities of anticancer activity. The MM-PBSA energy calculations support the strong binding of the potential lead molecules with the PKM2 protein. Based on the data, out the three hit compounds, we cannot be sure to label one compound as having the most potential, since one compound ranked better in one parameter, while other compounds were good in other properties. Hence, we propose Ver, Rut, and Lut as potential lead compounds for further study in the drug development process with the PKM2 protein against cancer.

## Figures and Tables

**Table 1:** List of bioactive compounds, their micro constituents and molecular formula present in olive leaf extract (OLE).

Compound Names	Micro Constituent	Formula	Reference
Rutin	Flavonoid	C27H30O16	[13]
Luteolin_7_*O*_glucoside	Flavonoid	C21H20O11	[14]
Verbascoside	Phenolic compound	C29H36O15	[13]
Maslinic acid	Triterpenoid	C30H48O4	[15]
Oleuropein	Secoiridoid	C25H32O13	[1]
Lucidumoside C	Secoiridoid	C27H36O14	[14]
Ursolic acid	Triterpenoid	C30H48O3	[13]
Oleanolic acid	Triterpenoid	C30H48O3	[13]
Uvaol	Phenolic compound	C30H50O2	[15]
Betulinic acid	Triterpenoid	C30H48O3	[16]
Oleoside	Secoiridoid	C16H22O11	[13]
Elenolic acid glucoside	Secoiridoid	C17H25O11	[13]
Loganic acid	Phenolic compound	C16H24O10	[17]
Diosmetin	Flavone glycoside	C21H13O11	[18]
Apigenin	Flavonoid	C15H10O5	[13,14]
Secologanin	Phenolic compound	C17H24O10	[17]
Ferulic acid	Phenolic compound	C10H10O4	[19]
Hydroxytyrosol	Phenolic compound	C8H10O3	[13]
Gallic acid	Phenolic compound	C7H6O5	[20]
Coumaric acid	Phenolic compound	C9H8O3	[21]
Homovanillyl alcohol	Phenolic compound	C9H12O3	[13]
Cinnamic acid	Phenolic compound	C9H8O2	[13]
Tyrosol	Phenolic compound	C8H10O2	[22]

**Table 2:** Assessment of the toxicity of different bioactive compounds of olive leaf extract (OLE) using pkCSM webserver.

Compound Names	AMES Toxicity	hERG-I Inhibitor	Hepato-Toxicity	Skin Sensitisation	*T. pyriformis* Toxicity (log µg/L)	Oral Rat Acute Toxicity (mol/kg)
Rutin	No	No	No	No	0.285	2.491
Verbascoside	No	No	No	No	0.285	2.527
Luteolin_7_*O*_glucoside	No	No	No	No	0.285	2.547
Ursolic acid	No	No	Yes	No	0.285	2.346
Oleanolic Acid	No	No	Yes	No	0.285	2.349
Oleuropein	No	No	No	No	0.285	2.862
Maslinic acid	No	No	Yes	No	0.285	2.516
Uvaol	No	No	Yes	No	0.376	2.646
Betulinic acid	No	No	Yes	No	0.285	2.256
Apigenin	No	No	No	No	0.38	2.45
Loganic acid	No	No	No	No	0.285	1.995
Oleoside	No	No	No	No	0.285	2.389
Diosmetin	No	No	No	No	0.336	2.338
Elenolic acid glucoside	No	No	No	No	0.285	2.35
Lucidumoside C	No	No	No	No	0.285	3.068
Secologanin	No	No	No	No	0.285	2.031
Coumaric acid	No	No	No	No	0.319	2.155
Cinnamic acid	No	No	No	No	0.247	2.094
Ferulic acid	No	No	No	No	0.271	2.282
Hydroxytyrosol	Yes	No	No	No	−0.128	1.858
Gallic acid	No	No	No	No	0.285	2.218
Tyrosol	No	No	No	Yes	−0.244	1.861
Homovanillyl alcohol	No	No	No	No	−0.059	1.891
	1/total	0/total	5/total	1/total		

*T. pyriformis* toxicity- Aquatic toxicity model. Oral Rat Acute Toxicity is presented as LD50.

**Table 3:** Evaluation of the ADME properties of the phytocompounds present in the olive leaf extract (OLE) using pkCSM web server for human model.

Compound Names	Absorption		Distribution	Metabolism	Excretion
	Water Solubility (log mol/L)	Intestinal Absorption (%)	Fraction Unbound (Fu)	CYP3A4 Inhibitor	Total Clearance (log mL/min/kg)
Rutin	−2.892	3.446	0.187	No	−0.369
Verbascoside	−2.906	32.119	0.269	No	0.479
Luteolin_7_*O*_glucoside	−2.716	37.556	0.224	No	0.478
Ursolic acid	−3.072	100	0	No	0.083
Oleanolic Acid	−3.074	99.931	0	No	0.285
Oleuropein	−2.722	44.206	0.485	No	1.176
Maslinic acid	−3.042	100	0.033	No	−0.071
Uvaol	−5.947	92.819	0	No	0.206
Betulinic acid	−3.122	99.763	0.018	No	0.116
Apigenin	−3.329	93.25	0.147	No	0.566
Loganic acid	−2.365	16.515	0.641	No	1.225
Oleoside	−2.604	0	0.567	No	1.395
Diosmetin	−3.238	79.898	0.068	No	0.598
Elenolic acid glucoside	−2.517	20.4	0.606	No	1.482
Lucidumoside C	−2.833	41.241	0.418	No	1.146
Secologanin	−2.676	40.54	0.611	No	1.585
Coumaric acid	−2.378	93.494	0.428	No	0.662
Cinnamic acid	−2.608	94.833	0.38	No	0.781
Ferulic acid	−2.817	93.685	0.343	No	0.623
Hydroxytyrosol	−1.139	72.809	0.593	No	0.23
Gallic acid	−2.56	43.374	0.617	No	0.518
Tyrosol	−1.146	85.255	0.485	No	0.283
Homovanillyl alcohol	−1.433	84.608	0.395	No	0.305

**Table 4:** Anti-cancer predictions of the phytocompounds present in the olive leaf extract (OLE) using Way2Drug (pass) server.

Compound Name	Pyruvate Kinase Inhibition		Antineoplastic	
	Pa	Pi	Pa	Pi
Rutin	0.229	0.009	0.849	0.007
Luteolin_7_*O*_glucoside	0.680	0.002	0.830	0.009
Verbascoside	0.115	0.017	0.814	0.010
Maslinic	--	--	0.867	0.005
Oleuropein	--	--	0.387	0.109
Lucidumoside C	--	--	0.674	0.030
Ursolic acid	--	--	0.857	0.006
Oleanolic acid	--	--	0.876	0.005
Uvaol	--	--	0.907	0.005
Betulinic acid	--	--	0.925	0.005
Oleoside	0.091	0.025	0.796	0.012
Elenolic acid glucoside	0.063	0.050	0.795	0.012
Loganic acid	0.071	0.040	0.817	0.010
Diosmetin	0.135	0.014	0.791	0.013
Apigenin	0.280	0.007	0.774	0.015
Secologanin	--	--	0.500	0.071
Ferulic acid	0.080	0.031	0.601	0.045
Hydroxytyrosol	0.119	0.016	--	--
Gallic acid	0.124	0.016	0.313	0.145
Coumaric acid	0.163	0.011	0.520	0.065
Homovanillyl alcohol	0.070	0.040	0.321	0.140
Cinnamic acid	0.141	0.013	0.455	0.085
Tyrosol	0.132	0.014	--	--

**Table 5:** Screening of the phytocompounds to obtain potential lead molecules (bold) based on their respective binding energy profile upon docking with PKM2 using Autodock vina.

Compound Names	PubChem CID	Binding Energy (kcal/mol)	Ki (µmol/L)	Binding Site
Rutin	5280805	−10	0.046	CS
Verbascoside	5281800	−9.8	0.064	CS
Luteolin_7_*O*_glucoside	5280637	−8.9	0.296	CS
Ursolic acid	64945	−8.9	0.296	OS
Oleanolic Acid	10494	−8.8	0.351	CS
Oleuropein	5281544	−8.8	0.351	CS
Maslinic	73659	−8.7	0.415	OS
Uvaol	92802	−8.7	0.415	OS
Betulinic acid	64971	−8.4	0.690	OS
Apigenin	5280443	−8.1	1.145	OS
Loganic acid	89640	−8	1.356	CS
Oleoside	101042548	−8	1.356	CS
Diosmetin	5281612	−9	0.250	CS
Elenolic acid glucoside	10692563	−7.6	2.665	CS
Lucidumoside C	10793430	−7.5	3.155	CS
Secologanin	161276	−7.1	6.201	CS
Coumaric acid	637542	−6.9	8.693	AS
Cinnamic acid	444539	−6.4	20.226	CS
Ferulic acid	445858	−6.3	23.947	OS
Hydroxytyrosol	82755	−6.3	23.947	AS
Gallic acid	370	−6	39.746	CS
Tyrosol	10393	−6	39.746	AS
Homovanillyl alcohol	16928	−5.7	65.968	CS

Ki: Inhibition content; AS: allosteric site, CS: catalytic site; OS: other site (other than catalytic and allosteric site).

**Table 6:** Binding free energy of ligands with PKM2 calculated by the MM-PBSA method for 100 snapshots of MD simulation.

	Ligands		
	Verbascoside	Rutin	Luteolin_7_O_Glucoside
**ΔE_vdW_**	−47.63 ± 6.78	−35.32 ± 2.91	−40.88 ± 5.98
**ΔE_ele_**	−39.57 ± 6.32	−40.47 ± 6.57	−26.22 ± 10.80
**ΔE_PSE_**	85.42 ± 11.35	77.30 ± 12.81	56.68 ± 17.23
**ΔES_SASA_**	−5.98 ± 0.39	−4.72 ± 0.44	−4.57 ± 0.50
**ΔE_BE_**	−7.76 ± 5.95	−3.22 ± 3.22	−15.00 ± 5.58

**ΔE_vdW_:** van der Waal energy, **ΔE_ele_:** Electrostatic energy, **ΔE_PSE_:** Polar solvation energy, **ΔE_SASA_:** Solvent accessible surface area energy, **ΔE_BE_:** Binding energy.

**Table 7:** Total binding energies (kcal mol^−1^) of major energy contributors for the interaction of ligands with PKM2 calculated from MM-PBSA.

Luteolin_7_O_Glucoside		Rutin		Verbascoside	
Residues	E_total_	Residues	E_total_	Residues	E_total_
Thr50	−0.71 ± 0.04	Thr328	−0.68 ± 0.06	Thr50	−0.72 ± 0.02
Pro53	−1.96 ± 0.04	Gln329	−0.80 ± 0.11	Pro53	−0.68 ± 0.02
Leu74	−0.63 ± 0.04	Ile335	−1.38 ± 0.07	Ser77	−0.59 ± 0.08
His78	−1.87 ± 0.09	Gly363	−0.61 ± 0.05	His78	−0.54 ± 0.05
Gly79	−0.52 ± 0.04	Glu364	−0.55 ± 0.25	Asp113	−0.81 ± 0.17
Tyr83	−2.20 ± 0.12			Thr114	−0.73 ± 0.06
Ala366	−0.77 ± 0.05			Glu118	−1.11 ± 0.05
				Val176	−0.60 ± 0.03
				Asp177	−0.96 ± 0.05
				Val209	−0.88 ± 0.02
				Glu332	−0.56 ± 0.08
				Ala366	−0.59 ± 0.03

**E_total_:** Total energy.

## Data Availability

The raw data of this manuscript is available by corresponding author upon request.

## Abbreviations

ADME	Absorption, distribution, metabolism, excretion
Lut	Luteolin_7_*O*_glucoside
OLE	Olive leaves extract
PKM2	Pyruvate kinase M2 isoform
Rut	Rutin
Ver	Verbascoside
